# Blood-Based Bioenergetic Profiling Reflects Differences in Brain Bioenergetics and Metabolism

**DOI:** 10.1155/2017/7317251

**Published:** 2017-10-02

**Authors:** Daniel J. Tyrrell, Manish S. Bharadwaj, Matthew J. Jorgensen, Thomas C. Register, Carol Shively, Rachel N. Andrews, Bryan Neth, C. Dirk Keene, Akiva Mintz, Suzanne Craft, Anthony J. A. Molina

**Affiliations:** ^1^Frankel Cardiovascular Center, Department of Internal Medicine, University of Michigan Medical School, Ann Arbor, MI 48109, USA; ^2^Section on Comparative Medicine, Department of Pathology, Wake Forest School of Medicine, Winston-Salem, NC 27157, USA; ^3^Section on Gerontology and Geriatrics, Sticht Center for Healthy Aging and Alzheimer's Prevention & Department of Internal Medicine, Wake Forest School of Medicine, Winston-Salem, NC 27157, USA; ^4^Department of Pathology, University of Washington, Seattle, WA, USA; ^5^Department of Radiology, Wake Forest School of Medicine, Winston-Salem, NC 27157, USA

## Abstract

Blood-based bioenergetic profiling provides a minimally invasive assessment of mitochondrial health shown to be related to key features of aging. Previous studies show that blood cells recapitulate mitochondrial alterations in the central nervous system under pathological conditions, including the development of Alzheimer's disease. In this study of nonhuman primates, we focus on mitochondrial function and bioenergetic capacity assessed by the respirometric profiling of monocytes, platelets, and frontal cortex mitochondria. Our data indicate that differences in the maximal respiratory capacity of brain mitochondria are reflected by CD14+ monocyte maximal respiratory capacity and platelet and monocyte bioenergetic health index. A subset of nonhuman primates also underwent [^18^F] fluorodeoxyglucose positron emission tomography (FDG-PET) imaging to assess brain glucose metabolism. Our results indicate that platelet respiratory capacity positively correlates to measures of glucose metabolism in multiple brain regions. Altogether, the results of this study provide early evidence that blood-based bioenergetic profiling is related to brain mitochondrial metabolism. While these measures cannot substitute for direct measures of brain metabolism, provided by measures such as FDG-PET, they may have utility as a metabolic biomarker and screening tool to identify individuals exhibiting systemic bioenergetic decline who may therefore be at risk for the development of neurodegenerative diseases.

## 1. Introduction

There is mounting evidence that blood-based respirometric profiling can report on systemic bioenergetic capacity. Previous studies link mitochondrial parameters measured in peripheral blood mononuclear cells (PBMCs), made up of monocytes and lymphocytes, and platelets to various age-related diseases and disorders such as AD, diabetes, and frailty [1–6]. Our previous studies have shown that the respirometric profiles of blood cells are related to features of aging that are associated with morbidity and mortality, including reduced physical ability and inflammation [7, 8]. More recently, we reported that blood cell respirometry reflects the bioenergetic capacity of highly metabolically active tissues such as skeletal and cardiac muscles [9]. These studies support potential diagnostic applications of minimally invasive, blood-based measures of mitochondrial function [10]. The goal of this study was to expand on this body of knowledge by investigating the relationships of blood cell respirometry to measures of brain bioenergetics and metabolism.

Highly metabolically active tissues are particularly susceptible to bioenergetic decline. The adult brain, which accounts for just ~2% of total body weight, utilizes ~20% of total body O_2_ consumption and ~60% of total body glucose and requires a daily energy input of ~420 kcal [11, 12]. This exceptionally high metabolic demand makes the brain remarkably sensitive to the deleterious effects of mitochondrial dysfunction. In 2004, Swerdlow and Khan proposed the “mitochondrial cascade hypothesis” for the development of sporadic late-onset AD, stating that mitochondrial dysfunction is the primary event leading to the deposition of senile plaques and neurofibrillary tangles that are hallmarks of this disease [13]. Over the past decade, it has been increasingly recognized that changes in mitochondrial function are apparent at the earliest presymptomatic stages of AD and related to the progression of disease [14]. Multiple studies link the deposition of amyloid-*β* (A*β*) to alterations in mitochondrial bioenergetics, for example, depolarization, uncoupling of the electron transport chain (ETC), reduced ATP production, and increased reactive oxygen species generation [15–18]. Studies of patients and animal models indicate that AD is associated with the accumulation of amyloid precursor protein (APP) and A*β* on brain mitochondria, leading to bioenergetic changes [19–24]. Greater AD risk is associated with reduced cerebral glucose metabolic rate, measured by [^18^F] fluorodeoxyglucose positron emission tomography (FDG-PET), which can appear years before dementia onset [25, 26]. Thus, FDG-PET has emerged as a powerful method for the early detection of AD and may help differentiate mild AD from other forms of dementia [27, 28]. A subset of primates utilized in this project underwent brain FDG-PET imaging, providing us with a unique opportunity to obtain preliminary data on the relationships between blood cell respirometry and brain glucose metabolism.

Multiple lines of evidence indicate that peripheral mitochondrial dysfunction accompanies changes in brain mitochondria in AD. Analysis of white blood cells from patients with early AD shows that the expression of mitochondrial respiratory complex I–V genes and subunits of the core mitochondrial ribosome complex are decreased compared to controls [29]. The authors report that these differences mirror changes observed in AD brains. Circulating lymphocytes from patients with AD also exhibit a pathological pattern of mitochondrial dysfunction and increased oxidative damage [30–32]. Platelet mitochondrial function has been shown to be impaired in patients with mild cognitive impairment (MCI) and AD compared to healthy age-matched controls [1, 33, 34]. Several groups are now exploring the use of blood cells for early diagnosis of AD [35–37]. The goal of this project is to expand on this growing body of knowledge by examining the relationships between brain bioenergetics and metabolism with CD14+ monocyte and platelet mitochondrial function.

First described in 1955, respirometry remains the gold standard assessment of mitochondrial function and is able to capture the cumulative effects of intrinsic and extrinsic factors on bioenergetic capacity [29–31]. In this study, we utilized mitochondrial respirometry to assess mitochondrial function in circulating cells and isolated brain (frontal cortex) mitochondria. The assessments were performed on nonhuman primates, representing various levels of cardiometabolic health across young and old age groups. This broad selection criterion was utilized in order to maximize potential differences in systemic bioenergetic capacity between animals. We tested the hypothesis that the respirometric profile of circulating cells reflects differences in systemic bioenergetic capacity, including differences in brain bioenergetics and metabolism. This hypothesis is based on the recognition that blood cells are continuously exposed to circulating factors such as inflammatory cytokines, redox stress [36], and recently described mitokines [37], which can affect mitochondrial function across different tissues and organ systems. Our goal is to examine blood-based bioenergetic profiling as a potential biomarker for changes in brain metabolism that has been linked to the development of neurodegenerative diseases such as AD.

## 2. Materials and Methods

### 2.1. Animals

Female vervet/African green monkeys (*Chlorocebus aethiops sabaeus*) ranging in age from 8.2 to 23.4 years were utilized for this study. Animals were socially housed in stable groups of 11–49 in housing units with indoor and outdoor access of approximately 28 m^2^ indoors and 111 m^2^ outdoors which contained perches, platforms, elevated climbing structures, and a base composed of smooth stones. Prior to the study, 7 of the 18 animals were moved to indoor housing (pair- or individually housed). All animals were fed with a standard monkey chow diet (Lab Diet 1538) that was supplemented with fruits and vegetables 5 times per week. Water was available ad libitum. Immediately prior to necropsy, blood samples were collected from anesthetized animals. Frontal cortex tissues were taken during necropsy.

### 2.2. Body Mass and Blood Glucose

Body weight was measured at the time of necropsy. Fasting glucose was determined via an autoanalyzer (ACE Alera) from Alfa Wassermann Diagnostic Technologies (West Caldwell, NJ). These analyses were performed in the Wake Forest Comparative Medicine Clinical Chemistry and Endocrinology Laboratory 4–12 months prior to necropsy.

### 2.3. Isolation of Blood Cells

Acid citrate dextrose (ACD) tubes (Vacutainer; Becton Dickinson, Franklin Lakes, NJ) were used to collect 8 ml of blood from fasted monkeys. Samples were maintained at room temperature and processed immediately to obtain platelet and CD14+ monocyte populations. The method for isolating platelets and CD14+ monocytes has been described [38]. Whole blood was centrifuged at 500 ×g for 15 minutes in ACD tubes with no brake at room temperature. Platelet rich plasma was removed and centrifuged at 1500 ×g for 10 minutes to isolate platelets which were washed in phosphate-buffered saline (PBS) with prostaglandin E_1_ (PGE1; Cayman Chemical, Ann Arbor, MI) and resuspended in extracellular flux (XF) assay buffer (Seahorse Biosciences, North Billerica, MA) containing 1 mM Na^+^ pyruvate, 1 mM GlutaMAX (Gibco, Grand Island, NY), 11 mM D-glucose, and PGE1 (pH 7.4). At the same time, the peripheral blood mononuclear cell (PBMC) layer was extracted, diluted in RPMI 1640 (Gibco), and layered onto 3 mL of polysucrose solution at a density of 1.077 g/mL (Sigma Histopaque®-1077, St. Louis, MO) in 15 mL centrifuge tubes. This was centrifuged at 700 ×g for 30 minutes with no brake to purify the PBMCs and remove red blood cells. The purified PBMC layer was obtained and washed in PBS. Finally, CD14+ monocytes were isolated using CD14-labeled magnetic microbeads (Miltenyi Biotec, San Diego, CA) according to the manufacturer's instructions using modified RPMI 1640 + fatty acid free bovine serum albumin (BSA) media. Platelets and monocytes were counted via a hemacytometer. Monocytes were washed again, using the same buffer, and resuspended in XF assay buffer without PGE1.

### 2.4. Respirometry of Blood Cells

Respirometry of blood cells was carried out using the Seahorse XF24-3 extracellular flux analyzer (Seahorse Bioscience, Agilent) with 250,000 monocytes and ~25,000,000 platelets in quadruplicate [39]. The methods of bioenergetic profiling have been described before [40]. Basal oxygen consumption rate (OCR) measures were monitored prior to any chemical additions. Then, sequential additions of oligomycin (750 nM), carbonyl cyanide-4-(trifluoromethoxy) phenylhydrazone (FCCP; 1 *μ*M), and antimycin A + rotenone (A/R; both 1 *μ*M) (all from Sigma-Aldrich) were added with measurements taken after each. FCCP stimulates the maximal OCR. The difference between the maximal OCR and basal OCR is termed the reserve capacity which has been reported on previously [41, 42]. The difference between basal and oligo is the ATP-linked OCR [43]. The difference between the oligomycin-induced respiration (oligo) and A/R is reported as leak respiration. The bioenergetic health index (BHI) was calculated according to the equation from Chacko et al. [38]. Monocyte respiration is reported as pmol/min/250,000 cells and platelet respiration was normalized to total protein content (mg), determined by Pierce BCA assay (Thermo Fisher Scientific, Grand Island, NY), and reported as pmol/min/mg protein.

### 2.5. FDG-PET Imaging

Animals were anesthetized with ketamine for transfer to the imaging suite. Animals were then intubated and maintained on isoflurane for the duration of the imaging. An indwelling venous catheter was introduced into the saphenous vein for continuous blood sampling during scan acquisition and a second catheter placed in the contralateral saphenous vein for the injection of 5 mCi of tracer. Blood samples were obtained at 3, 8, 16, 24, 35, and 55 min after FDG infusion.

^18^F-FDG-PET imaging used the time frames 12 × 10 sec, 8 × 30 sec, 6 × 4 min, and 3 × 10 min, for a total length of 60 min. PET images were preprocessed and coregistered to T1-weighted anatomical magnetic resonance (MR) images using a cross-modality 3D image fusion tool in PMOD 3.5. MR images were acquired on a 3 T Siemens Skyra MRI scanner using a 3D volumetric MPRAGE sequence (TR = 2700 msec; TE = 3.39 msec; TI = 880 msec; FA = 8 degrees; and 160 slices, voxel dimension = 0.5 × 0.5 × 0.5 mm). Coregistered PET images were corrected for partial-volume effect with the modified Müller-Gartner method. Using the PMOD 3.5 pixel-wise kinetic modeling tool, parametric images of cerebral metabolic rate of glucose (CMRg) were produced. CMR quantification included an arterial input function, determined by tracing regions of interest on the internal carotid arteries with the aid of coregistered MR images. The lumped constant used for the CMRg calculation was set to 0.344.

The CMR data for this study were processed with two atlases. Data for components of the vervet anterior cingulate cortex (Brodmann areas 24, 25, and 32), amygdala, and the anterior/posterior hippocampus were defined with methods described in [44]. Data for the frontal cortex were defined from a well-established nonhuman primate atlas, UNC-parcellation [45]. Complete methods for regional CMR generation with the two atlases can be found at Maldjian et al. 2015 [46].

### 2.6. Euthanasia

Ketamine (10–15 mg/kg, IM) followed by sodium pentobarbital (60–100 mg/kg, IV) was the method of euthanasia used to attain deep surgical anesthesia followed by exsanguination in accordance with guidelines established by the Panel on Euthanasia of the American Veterinary Medical Association. All procedures were approved and performed according to the guidelines of state and federal laws, the US Department of Health and Human Services, and the Animal Care and Use Committee of Wake Forest School of Medicine.

### 2.7. Isolation of Mitochondria from the Frontal Cortex

Methods of isolating mitochondria have been described previously and were modified slightly for frontal cortex samples [47]. For each animal, ~50 mg of tissue was minced into small pieces and resuspended in Chappell-Perry buffer I (CP I) containing ~1 mg/ml Nagarse (Sigma-Aldrich) for 5 minutes at room temperature. Frontal cortex samples were homogenized by 6 strokes using a glass-on-glass dounce homogenizer. The homogenate was washed with an equal volume of CP I and 2x volume of CP II buffer by centrifuging at 600 ×g at 4°C for 10 minutes (Eppendorf Centrifuge 5804R, Eppendorf AG, Hamburg, Germany). The supernatant was then filtered through cheese cloth and subsequently centrifuged at 10,000 ×g at 4°C for 10 minutes using a Beckman centrifuge, model J2-21 M Induction drive centrifuge (Beckman-Coulter, Inc., Brea, CA). The mitochondrial pellet was washed twice more with the same conditions then resuspended in mitochondrial assay solution (mannitol and sucrose buffer: MAS; sucrose 35 mM, mannitol 110 mM, KH_2_PO_4_ 2.5 mM, MgCl_2_ 2.5 mM, HEPES 1.0 mM, EGTA 0.5 mM, fatty acid free BSA 0.10%, and pH 7.4) prior to respirometry. The protein content of the purified frontal cortex mitochondria was determined by Pierce BCA assay (Thermo Fisher Scientific, Grand Island, NY). From 50 mg of tissue, ~500 *μ*g of purified mitochondria was obtained.

### 2.8. Respirometry of Isolated Mitochondria

Respirometry of frontal cortex-isolated mitochondria was performed using the XF24-3 as previously described in other tissues [47, 48]. This protocol was optimized for respiration driven by complex 2, which provides consistently higher values in accordance with previous reports [48]. Chemical additions were prepared in 1x MAS at 10x the final concentration required (2 mM ADP, 2 *μ*M oligomycin, 6 *μ*M FCCP, and 2 *μ*M antimycin A). Isolated mitochondria (5 *μ*g) were plated in quadruplicate in 50 *μ*l and attached via centrifugation at 2000 ×g at 4°C for 20 minutes. After centrifugation, 450 *μ*l of 1x MAS containing succinate (10 mM) and rotenone (2 *μ*M) was gently added to each well to a total volume of 500 *μ*l. The respirometric profiling procedure first stimulates maximal State 3 respiration with ADP, followed by inhibition of ATP synthase with oligo, providing the State 4o. Then, FCCP is injected which uncouples the electron transport chain to induce another measure of maximal respiration. Finally, antimycin A is titrated to block the flow of electrons at complex III and halt respiration. The respiration of isolated mitochondria is reported as pmol/min/5 *μ*g mitochondrial protein.

### 2.9. Statistical Analyses

Shapiro-Wilk tests ensured that all variables were normally distributed. Pearson correlation coefficients were assessed between all variables, and partial correlations for age, weight, and glucose level were also assessed. Analyses of monocyte and platelet respirometry were focused on maximal uncoupled respiration induced by FCCP, reserve capacity (calculated by subtracting basal from maximal respiration), and BHI. In order to provide a corresponding measure from contemporaneous respirometric profiling of brain mitochondria, analyses of frontal cortex mitochondrial respiration were focused on maximal uncoupled respiration induced by FCCP. Analyses of brain metabolism by FDG-PET focused on multiple brain regions in order to account for potential regional differences. Significance was set at an *α*-level of 0.05. The analyses were performed (SPSS v22; Armonk, NY).

## 3. Results

### 3.1. Characterization of Primates

Female vervets were selected to represent a wide range of metabolic health status as evidenced by insulin resistance as well as body mass indices from lean to obese across young and old age groups. Age, body weight, and fasting glucose are summarized in Table 1. A total of 15 vervets were studied with a mean age of 15.2 years. Ages ranged from 8.2 to 23.4 years. The mean weight of the animals was 4.8 kg, ranging from 3.3–6.9 kg. Fasting plasma glucose level averaged 128.6 mg/dL, ranging from 61–319 mg/dL. Bioenergetic characteristics of monocytes, platelets, and isolated brain mitochondria are tabulated in Table 2. Large standard deviations reflect the intended diversity of our cohort.

### 3.2. Pearson and Partial Correlations between Monocyte/Platelet Respiration and the Bioenergetic Capacity of Mitochondria Isolated from the Frontal Cortex Tissue

**Figure 1:**
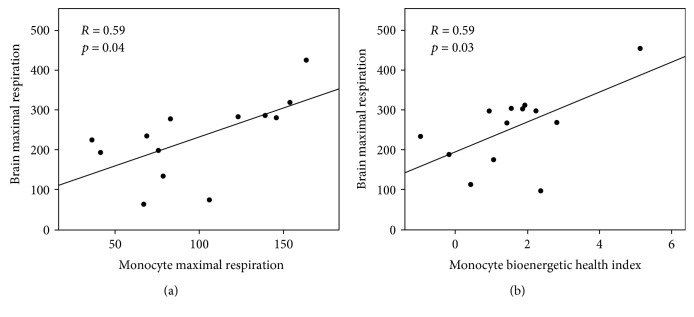
Associations of CD14+ monocyte (a) maximal respiration (pmol/min/250,000 cells) and (b) BHI with the maximal respiratory capacity of frontal cortex mitochondria (pmol/min/5 *μ*g mitochondrial protein). Pearson's correlations and *p* values for each association are shown.

Pearson correlation coefficients were used to compare blood cell bioenergetic parameters with brain-isolated mitochondrial respiration. The relationships between monocyte respiratory parameters (maximal OCR, reserve capacity, and OCR) and the maximal respiration of mitochondria isolated from the frontal cortex tissue are summarized in Table 3. Representative regression plots are shown in Figure 1. Maximal FCCP-linked respiration in monocytes was significantly positively correlated with maximal FCCP-linked respiration measured from brain mitochondria (*R* = 0.59, *p* = 0.04). This relationship remained significant even when controlling for age, body weight, and plasma glucose concentration. Similar results are observed when comparing monocyte reserve capacity to brain mitochondria; however, statistical significance was only evident when controlling for glucose (*R* = 0.65, *p* = 0.02). BHI was significantly positively correlated with brain mitochondrial respiration (*R* = 0.59, *p* = 0.03). This relationship was maintained when controlling for glucose; however, only trends are maintained when controlling for age and body weight.

**Figure 2:**
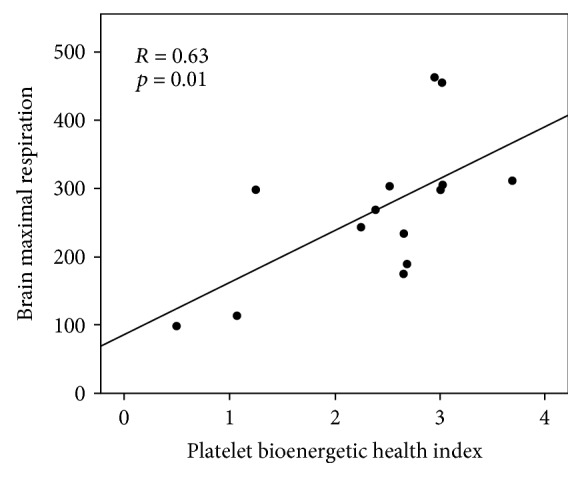
Association of platelet BHI with the maximal respiratory capacity of frontal cortex mitochondria (pmol/min/5 *μ*g mitochondrial protein). Pearson's correlation and *p* value are shown.

Relationships between platelet respiratory parameters and the maximal respiration of mitochondria isolated from the frontal cortex tissue are summarized in Table 4. A representative regression plot is shown in Figure 2. For platelets, BHI was significantly positively correlated with brain mitochondrial maximal respiration (*R* = 0.63, *p* = 0.01), even when controlling for age, body weight, and plasma glucose concentration. Interestingly, maximal OCR and reserve capacity were not associated.

### 3.3. Pearson and Partial Correlations between Monocyte/Platelet Respiration and Brain Glucose Metabolism

A subset of 5 animals was analyzed for both CD14+ respiration and brain metabolism by FDG-PET (summarized in Table 5). A subset of 7 animals was analyzed for both platelet respiration and brain metabolism (summarized in Table 6). Strong trends for positive correlations between monocyte BHI and FDG-PET were observed when controlling for age; statistical significance was reached for the amygdala and frontal cortex regions (*R* = 0.96, *p* = 0.04; *R* = 0.96, *p* = 0.04; resp.). For platelets, reserve respiratory capacity and BHI were positively correlated with FDG-PET measures in all brain areas examined. Strong trends are apparent throughout, and statistically significant relationships were observed between reserve capacity with the anterior cingulate cortex (Brodmann area 32), amygdala, and anterior hippocampus (*R* = 0.80, *p* = 0.03; *R* = 0.79, *p* = 0.04; *R* = 0.78, *p* = 0.04; resp.). These relationships remained statistically significant when controlling for body weight.

## 4. Discussion

Blood-based bioenergetic profiling has been proposed to be a minimally invasive indicator of mitochondrial health [9, 38]. While respirometric profiling of circulating cells is now being utilized in various human studies, little is known about how these measures relate to the bioenergetic capacity of various tissues of interest. Using a nonhuman primate model, we recently reported that the bioenergetic capacity of circulating cells is significantly positively related to skeletal and cardiac muscle bioenergetics. In this report, we focus on the brain, the organ with the highest metabolic demand. We show for the first time significant positive correlations between contemporaneous blood cell and brain mitochondrial respirometry. Furthermore, brain glucose metabolism assessed by FDG-PET imaging was similarly positively associated with the bioenergetic profile of circulating blood cells.

Mitochondrial dysfunction is apparent in the pathophysiology of various diseases and is widely recognized to be a potential target for intervention. Bioenergetic deficits may be the result of genetic abnormalities, acute disruptions, or accumulated damage as is the case with chronic diseases, including those associated with aging. Neuronal mitochondrial dysfunction is particularly damaging, likely due to the high metabolic demands of the brain. Numerous studies have linked mitochondrial alterations to the development of AD. Neuronal A*β* has been shown to directly interact with mitochondria to inhibit complex IV of the ETC [49]. Alterations in mitochondrial quality control processes, such as mitochondrial fusion, fission, and autophagy, are also associated with neurodegeneration and specifically to AD [50–57]. Specific mitochondrial DNA (mtDNA) mutations are related to cognitive function, AD status, and risk [58–62]. These alterations can all contribute to bioenergetic decline and alterations in brain metabolism associated with AD. As a result, measures of brain metabolism are currently being utilized to study the pathophysiology of AD and are widely recognized to have important diagnostic implications. The study reported here provides the first evidence suggesting that brain mitochondrial metabolism may be related to the bioenergetic profiles of blood cells.

Previous studies employing blood cell respirometry have focused on heterogeneous cell populations such as mixed peripheral blood mononuclear cells (PBMCs). In order to avoid the potential confounding effects of changes in PBMC cellular composition, this study focused on purified CD14+ monocytes. It is notable that a recent large-scale study of CD14+ monocytes from 1264 individuals reported that networks of genes related to oxidative phosphorylation and mitochondrial protein synthesis were differentially expressed based on chronological age [63]. We also performed respiratory analyses on platelets which are readily available and easy to isolate. Several groups are already exploring the potential utility of platelets in the diagnosis of diseases such as AD. Moreover, a recent study has reported that platelet bioenergetic capacity is related to AD status [34].

Blood-based bioenergetic profiling is not a surrogate for direct measures of brain metabolism. Rather, it may serve as a cost-effective screening tool to identify patients who may be more likely to exhibit neuronal bioenergetic deficits, based on systemic changes in mitochondria function. Our results indicating that blood-based bioenergetic profiling is related to FDG-PET measures of brain glucose metabolism were obtained from a small subset of primates and should be considered preliminary. A large-scale study is underway to confirm these findings in human subjects.

Our predetermined selection criteria were focused on maximizing differences in age and cardiometabolic health between animals. Hence, we include analyses controlling for age, body weight, and blood glucose in this report. Our sample size is not adequate to study the individual or interactive effects of each of these parameters. As designed, differences in cardiometabolic health were apparent across our younger as well as older animals. Indeed, comparing body weight and blood glucose between younger and older animals showed no statistical significance and a high level of variability. Larger scale future studies are required in order to determine how age, obesity, and insulin sensitivity/resistance are individually related to blood and brain bioenergetics.

## 5. Conclusions

Our data provide evidence that blood-based bioenergetic profiling can serve as a minimally invasive measure of systemic bioenergetic capacity that is positively related to measures of brain mitochondrial function and metabolism.

## Figures and Tables

**Table 1:** Demographics: *Chlorocebus aethiops sabaeus.*

*N* = 15	Mean	Range	SD
Age (yrs)	15.2	8.2–23.4	6.20
Weight (kg)	4.8	3.3–6.9	0.88
Fasting glucose (mg/dL)	128.6	61–319	70.31

**Table 2:** Respiratory parameters for monocytes, platelets, and isolated brain mitochondria.

	Mean	SD
*Monocyte respiration* (pmol/min/250,000 cells)		
Basal	50.64	20.82
Maximal	98.65	42.80
Leak	6.25	9.30
Nonmitochondrial	22.08	14.02
Reserve	48.02	27.56
Bioenergetic health index	1.58	1.50
*Platelet respiration* (pmol/min/mg protein)		
Basal	210.04	87.71
Maximal	419.93	161.31
Leak	17.95	28.38
Nonmitochondrial	85.92	20.76
Reserve	209.88	88.46
Bioenergetic health index	2.40	0.85
*Brain-isolated mitochondria* (pmol/min/5 *μ*g mitochondrial protein)		
State 3	250.31	112.86
State 4o	66.01	46.83
Maximal FCCP-linked	268.42	102.87
Nonmitochondrial	22.58	42.50

Monocyte: *N* = 13; platelet: *N* = 15; isolated brain mitochondria: *N* = 15.

**Table 3:** Pearson and partial correlations between CD14+ monocyte and isolated brain mitochondrial respiration.

Frontal cortex mitochondria: maximal respiration	Maximal		Reserve		BHI	
	*R*	*p*	*R*	*p*	*R*	*p*
Pearson:	**0.59**	**0.04**	0.49	0.09	**0.59**	**0.03**
*Partial correlations*						
Age	**0.62**	**0.03**	0.56	0.06	0.43	0.16
Body weight	**0.62**	**0.03**	0.56	0.06	0.42	0.17
Plasma glucose	**0.65**	**0.02**	**0.65**	**0.02**	**0.58**	**0.05**

**Table 4:** Pearson and partial correlations between platelet and isolated brain mitochondrial respiration.

Frontal cortex mitochondria: maximal respiration	Maximal		Reserve		BHI	
	*R*	*p*	*R*	*p*	*R*	*p*
Pearson	−0.34	0.21	−0.05	0.86	**0.63**	**0.01**
*Partial Correlations*						
Age	−0.28	0.33	−0.01	0.98	**0.67**	**0.01**
Body weight	−0.33	0.25	−0.10	0.73	**0.58**	**0.03**
Plasma glucose	−0.31	0.28	−0.00	0.99	**0.65**	**0.01**

**Table 5:** Pearson and partial correlations between monocyte respiration and brain glucose metabolism (FDG-PET).

FDG-PET: brain region	Maximal		Reserve		BHI	
	*R*	*p*	*R*	*p*	*R*	*p*
*Pearson*						
Area 24	0.46	0.44	0.26	0.67	0.40	0.50
Area 25	0.39	0.51	0.19	0.75	0.41	0.50
Area 32	0.50	0.39	0.28	0.64	0.43	0.46
Amygdala	0.42	0.48	0.23	0.71	0.28	0.65
Ant. hippocampus	0.46	0.44	0.31	0.61	0.26	0.68
Post. hippocampus	0.39	0.52	0.27	0.66	0.18	0.77
Frontal cortex	0.32	0.59	0.06	0.93	0.37	0.55
*Partial for age*						
Area 24	0.46	0.54	0.16	0.84	0.92	0.08
Area 25	0.38	0.62	0.08	0.92	0.89	0.11
Area 32	0.50	0.50	0.19	0.81	0.93	0.07
Amygdala	0.45	0.55	0.08	0.92	**0.96**	**0.04**
Ant. hippocampus	0.49	0.51	0.20	0.80	0.91	0.09
Post. hippocampus	0.42	0.58	0.13	0.87	0.89	0.11
Frontal cortex	0.31	0.69	−0.12	0.88	**0.96**	**0.04**
*Partial for weight*						
Area 24	0.11	0.89	0.04	0.96	0.36	0.64
Area 25	0.05	0.95	−0.02	0.98	0.36	0.64
Area 32	0.16	0.84	0.06	0.94	0.40	0.60
Amygdala	−0.07	0.93	−0.07	0.93	0.20	0.80
Ant. hippocampus	0.07	0.93	0.10	0.90	0.17	0.83
Post. hippocampus	−0.03	0.97	0.04	0.96	0.07	0.93
Frontal cortex	−0.26	0.74	−0.35	0.65	0.32	0.68
*Partial for plasma glucose*						
Area 24	0.46	0.54	0.28	0.72	0.41	0.59
Area 25	0.40	0.61	0.21	0.79	0.41	0.59
Area 32	0.51	0.50	0.31	0.69	0.45	0.56
Amygdala	0.46	0.55	0.32	0.68	0.33	0.68
Ant. hippocampus	0.47	0.53	0.36	0.64	0.27	0.73
Post. hippocampus	0.41	0.59	0.33	0.67	0.20	0.80
Frontal cortex	0.39	0.61	0.18	0.82	0.45	0.55

Areas 24, 25, and 32 = Brodmann areas (anterior cingulate cortex); Ant. = anterior; Post. = posterior; *N* = 5.

**Table 6:** Pearson and partial correlations between platelet respiration and brain glucose metabolism (FDG-PET).

FDG-PET: brain region	Maximal		Reserve		BHI	
	*R*	*p*	*R*	*p*	*R*	*p*
*Pearson*						
Area 24	0.55	0.20	0.72	0.07	0.74	0.06
Area 25	0.50	0.25	0.67	0.10	0.73	0.06
Area 32	0.65	0.11	**0.80**	**0.03**	**0.75**	**0.05**
Amygdala	0.67	0.10	**0.79**	**0.04**	0.70	0.08
Ant. hippocampus	0.64	0.12	**0.78**	**0.04**	0.74	0.06
Post. hippocampus	0.59	0.16	0.73	0.06	0.72	0.07
Frontal cortex	0.56	0.19	0.68	0.09	0.58	0.17
*Partial for age*						
Area 24	0.47	0.35	0.67	0.14	0.70	0.12
Area 25	0.41	0.42	0.62	0.19	0.69	0.13
Area 32	0.57	0.24	0.76	0.08	0.70	0.12
Amygdala	0.54	0.27	0.71	0.11	0.58	0.22
Ant. hippocampus	0.51	0.30	0.71	0.12	0.65	0.17
Post. hippocampus	0.43	0.39	0.64	0.17	0.60	0.21
Frontal cortex	0.45	0.38	0.60	0.21	0.47	0.35
*Partial for weight*						
Area 24	0.66	0.15	0.79	0.06	0.76	0.08
Area 25	0.60	0.21	0.74	0.10	0.74	0.09
Area 32	0.72	0.11	**0.83**	**0.04**	0.75	0.09
Amygdala	0.72	0.10	**0.81**	**0.05**	0.70	0.12
Ant. hippocampus	0.70	0.12	**0.81**	**0.05**	0.74	0.09
Post. hippocampus	0.66	0.16	0.77	0.08	0.72	0.11
Frontal cortex	0.66	0.15	0.74	0.09	0.58	0.23
*Partial for plasma glucose*						
Area 24	0.57	0.25	0.73	0.10	0.74	0.09
Area 25	0.50	0.31	0.68	0.14	0.73	0.10
Area 32	0.65	0.16	0.80	0.06	0.75	0.09
Amygdala	0.68	0.14	0.80	0.06	0.71	0.12
Ant. hippocampus	0.64	0.17	0.78	0.07	0.74	0.09
Post. hippocampus	0.60	0.21	0.74	0.09	0.72	0.11
Frontal cortex	0.58	0.22	0.72	0.11	0.60	0.21

Areas 24, 25, and 32 = Brodmann areas (anterior cingulate cortex); Ant. = anterior; post. = posterior; *N* = 7.
